# Decoding a highly mixed Kazakh genome

**DOI:** 10.1007/s00439-020-02132-8

**Published:** 2020-02-19

**Authors:** Madina Seidualy, Asta Blazyte, Sungwon Jeon, Youngjune Bhak, Yeonsu Jeon, Jungeun Kim, Anders Eriksson, Dan Bolser, Changhan Yoon, Andrea Manica, Semin Lee, Jong Bhak

**Affiliations:** 1 Korean Genomics Center (KOGIC), Ulsan National Institute of Science and Technology (UNIST), Ulsan, 44919 Republic of Korea; 2 Department of Biomedical Engineering, School of Life Sciences, Ulsan National Institute of Science and Technology (UNIST), Ulsan, 44919 Republic of Korea; 3 Personal Genomics Institute (PGI), Genome Research Foundation, Cheongju, 28160 Republic of Korea; 4 Department of Medical and Molecular Genetics, King’s College London, London, SE1 9RT UK; 5 cGEM, Institute of Genomics, University of Tartu, Riia 23b, 51010 Tartu, Estonia; 6 Geromics Ltd, Office 261, 23 Kings Street, Cambridge, CB1 1AH UK; 7 Department of Zoology, University of Cambridge, Downing Street, Cambridge, CB2 3EJ UK; 8 Clinomics LTD, Ulsan National Institute of Science and Technology (UNIST), Ulsan, 44919 Republic of Korea

## Abstract

We provide a Kazakh whole genome sequence (MJS) and analyses with the largest comparative Kazakh genomic data available to date. We found 102,240 novel SNVs and a high level of heterozygosity. ADMIXTURE analysis confirmed a significant proportion of variations in this individual coming from all continents except Africa and Oceania. A principal component analysis showed neighboring Kalmyk, Uzbek, and Kyrgyz populations to have the strongest resemblance to the MJS genome which reflects fairly recent Kazakh history. MJS’s mitochondrial haplogroup, J1c2, probably represents an early European and Near Eastern influence to Central Asia. This was also supported by the heterozygous SNPs associated with European phenotypic features and strikingly similar Kazakh ancestral composition inferred by ADMIXTURE. Admixture (*f3*) analysis showed that MJS’s genomic signature is best described as a cross between the Neolithic East Asian (Devil’s Gate1) and the Bronze Age European (Halberstadt_LBA1) components rather than a contemporary admixture.

**Electronic supplementary material:**

The online version of this article (10.1007/s00439-020-02132-8) contains supplementary material, which is available to authorized users.

## Introduction

Recently, a wide variety of genome sequencing technologies have become available heralding a new era of personal genomics (Lander et al. 2001) and many large population genome projects have been carried out. These include the 1000 Genomes Project (Abecasis et al. 2010), the UK’s 10,000 and 100,000 Genomes Projects (Walter et al. 2015; Samuel and Farsides 2017), the Genome of the Netherlands (Boomsma et al. 2014), the Estonian Biocentre’s Human Genome Diversity Panel (EGDP) (Pagani et al. 2016), the Simons Genome Diversity Project (SGDP) (Mallick et al. 2016), the Genome Russia project (Oleksyk et al. 2015), 1070 Japanese genomes (Nagasaki et al. 2015), and the Korean Reference (KOREF) and variome projects (Cho et al. 2016; Kim et al. 2018). The Personal Genome Project (PGP) (Ball et al. 2012) is perhaps the largest genome project in terms of openness and inclusiveness and aims to map all personal and ethnic genomes. However, there remain many practical issues for mapping and accurately analyzing all ethnic groups worldwide. One problem is suitable representation of highly admixed genomes (Medina-Gomez et al. 2015; Guryev 2017). Although the 1000 Genomes Project database has been expanding by adding more ethnic representatives, it currently contains only 2504 individuals from 26 populations (phase 3) and lacks much ethnic diversity including an absence of genomes from Central Asian populations (Sudmant et al. 2015). Other initiatives such as the SGDP and the EGDP include only a small number of Central Asian population representatives (Pagani et al. 2016; Mallick et al. 2016). Central Asian populations can be good targets for adding highly admixed samples to our knowledge base of the major and relatively homogeneous ethnic groups. Among many Central Asian countries, Kazakhstan is at the border of ethnically European and Asian nations (Mostafa 2013). Therefore, demographic inference from Kazakh whole genomes is of special value. We can use Kazakh genomic data as an independent line of evidence that complements inference from archeological and written histories, to understand the roots of the diverse phenotypic features and relationships with other populations. Despite the recently growing scientific interest, there is little Kazakh genomic data available (Pagani et al. 2016), including, few high coverage sequences with genome-wide genotype array data of 18 individuals (Pagani et al. 2016; Jeong et al. 2019). This data paucity causes insufficient description on the complexity of the Kazakh genetic structure and the demographic past of Central Asian populations.

Kazakhstan extends from the Caspian Sea on the west side to the Altai Mountains on the east and shares borders with Russia, China, Uzbekistan, Kyrgyzstan, and Turkmenistan. It is located at the crossroads of the Silk Road trade routes (Comas et al. 1998). Owing to multiple invasions throughout history, Kazakh territory has been the home to many distinct tribes and clans since the Paleolithic period (Ikawa-Smith 1978). Indo-European nomadic populations, namely Scythians-Saka started to settle in the Central Asian steppes at the beginning of the first millennium before the Common Era (BCE) (David Llewelyn Snellgrove 2018). Later on, and until the fourth century CE (370–452), a powerful state formed by the Huns prospered in the region of modern Kazakhstan, causing its inhabitants to move westward (Sinor 1990). Following the invasion of the Turkic-speaking tribes, Gokturk khanate was formed in the beginning of the sixth century CE (West 2009). The conquest of southern Kazakhstan by the Arabs followed by the invasion of Mongol tribes into the region led to increased societal complexity (Gibb 1923; Morgan 2007).

The current primary ethnic group of Kazakhstan, the Kazakhs, formed from the union of diverse tribal groups, namely Turkic (West 2009), Mongol (Morgan 2007), Huns (Sinor 1990), Nogays (Weissleder 1978), Iranians (David Llewelyn Snellgrove 2018), and Arabs (Gibb 1923). Furthermore, as a consequence of complicated historical events, Kazakhs have been territorially divided into three main hordes (Zhuz) since the fifteenth–sixteenth century CE. The Senior Zhuz tribes settled in eastern and southeastern Kazakhstan (Semirechye), the Middle Zhuz populated central Kazakhstan, and the Junior Zhuz lived primarily in western Kazakhstan. The Senior Zhuz are divided into eleven tribal groups, the Middle Zhuz into seven, and the Junior Zhuz into three (Olcott 1995). Traditionally marriage within the same tribe is undesirable (Forde 1934). Consequently, this has led to extensive mixing of Kazakh genomes. In this study, we aimed to increase the Asian PGP repertoire by providing the Kazakh (MJS’s) whole genome sequence; therefore, this study was carried out in the framework of the Pan Asian Population Genomics Initiative (PAPGI, http://papgi.org). We use this genome to confirm a high level of heterozygosity and attribute certain genomic components to both ancient and recent admixtures.

## Materials and methods

### Sample preparation

Our sample donor, MJS, is a healthy Kazakh female, who resides in southern Kazakhstan. MJS belongs genetically to two tribes: her father’s clan is a Middle Zhuz’s tribe, Naiman, people who migrated from Mongolia to settle in the eastern and central part of Kazakhstan in the late twelfth century CE (Akerov 2016); her mother’s clan is the Senior Zhuz’s tribe, Bayis, descendants of one of the major tribes of Dulat, people who settled in the southeast part (Semirechye) of Kazakhstan during the sixth and seventh centuries CE (Olcott 1995).

MJS reported her Kazakh ethnicity by providing a genealogical history of four generations. DNA was extracted from MJS’s peripheral blood using DNeasy Blood & Tissue Kit from QIAGEN according to the manufacturer’s protocols. Whole genome sequencing was conducted using the short-read sequencer, Illumina HiSeq X Ten, with 151 bp paired-end reads.

### Identification of individual variants

We used the GRCh37/hg19 (UCSC's nomenclature) as a reference. Before alignment of the reads to the reference, quality filtering was performed using NGSQC toolkit (v 2.3.3) with default options (Patel and Jain 2012). We used the Burrows–Wheeler Alignment-MEM (BWA v0.7.8) (Li and Durbin 2009) with a minimum seed length of 19 bp for mapping against the reference. Quality check of the mapping results was performed with SAMStat (v1.5.1) (Lassmann et al. 2011). The alignment file was sorted using the SAMtools (v. 0.1.19) (Li et al. 2009). Reads duplicated in PCR were removed using the MarkDuplicate option in Picard (v1.114) (http://broadinstitute.github.io/picard/). Local realignment of reads around indels and recalibration of base quality scores were performed using IndelRealigner and BaseRecalibrator in the Genome Analysis Toolkit (GATK v2.3.9) (McKenna et al. 2010). We used a GATK Unified Genotyper with the settings ‘-heterozygosity 0.0010-dcov 200-stand_call_conf 30.0-stand_emit_conf 30.0’ to call variants.

### Annotation of the variants

Single nucleotide variants (SNVs) and small insertions and deletions (indels) ranging from one to 20 bases were identified using GATK (McKenna et al. 2010). To annotate the type and functional consequences of SNVs and indels we used the snpEff (v4.3) (Cingolani et al. 2012) and ANNOVAR (v3) software (Wang et al. 2010). SNVs in the MJS genome were examined for their possible functional effects using computational prediction methods using SIFT (Ng and Henikoff 2003), Polyphen2 (Jordan et al. 2011), and PROVEAN (Choi and Chan 2015). We classified the variants into known and novel SNVs according to their presence in the dbSNP reference collection (https://www.ncbi.nlm.nih.gov/snp) (v147). Known variants were further annotated with possible associations to known diseases or drug responses using the databases OMIM (www.omim.org) and ClinVar (v20170130) (Landrum et al. 2014). The non-synonymous SNVs in MJS that were predicted to be functionally damaging were also checked in the other 21 publicly available Kazakh genomes (https://www.geenivaramu.ee/en), (Jeong et al. 2019). Due to limited SNP genotyping array coverage of the 18 samples, all of the selected SNVs (except for the rs1805124) were reported using only four Kazakh samples (Table S1).

### Admixture analysis

We examined heterogeneous admixture patterns using the ADMIXTURE (v1.3.0) (Alexander et al. 2009) program. We collected all the publically available genomic Kazakh data to date which reflected admixed Kazakh individuals in different tribes. Three Kazakh samples were obtained from the Estonian Genome Centers’ biobank (https://www.geenivaramu.ee/en); one from Central-West, one from Tien-Shan (southeastern Kazakhstan) and one from unidentified location in Kazakhstan, and 18 SNP chip based samples were obtained from the recently published data (Jeong et al. 2019) deposited in Max Plank digital library. Firstly, we merged the human origin SNP panel (HOSP) data containing 2345 samples from 203 populations worldwide (Lazaridis et al. 2014) with the sample dataset from the Estonian Genome Centers’ biobank (https://www.geenivaramu.ee/en), MJS’s genome and the 18 Kazakh dataset using PLINK (v1.90) (Purcell et al. 2007), utilizing autosomal SNPs. We pruned the panel with linkage disequilibrium (LD) using PLINK (v1.90) using the ‘-indep-pairwise 200 25 0.4’ option. We explored the values of the assumed ancestral populations (*K*) from two to 14. We observed the cross validation error values (Fig. S1) and chose *K* = 2, 4, 6, and 8 to display the increasing Kazakh ancestral complexity along with increasing *K* values. We also used qpGraph of ADMIXtool (Patterson et al. 2012) and 110 modern human genomes (French, Mongolian, Koryak, Yoruban) from the Human Origin SNP Panel (HOSP) (Lazaridis et al. 2014) to validate ADMIXTURE graphs.

### Mitochondrial haplogroup analysis

Variants in the mtDNA sequence were detected by mapping to the rCRS (revised Cambridge Reference Sequence of the human mtDNA) (Andrews et al. 1999). We used HaploGrep (v 2.1.13) to determine the haplogroup of MJS’s maternal lineage (Weissensteiner et al. 2016).

### Principal component analysis (PCA)

MJS genome was projected onto the first two principal components calculated using samples from PAPGI (http://papgi.org), HOSP (Lazaridis et al. 2014), and EGDP (http://evolbio.ut.ee/). To optimize the dataset and reduce bias caused by closely linked variants we pruned the merged dataset with LD using PLINK (v1.90) (Purcell et al. 2007) with the ‘–indep-pairwise 200 25 0.4’, ‘-geno 0.1’, ‘-maf 0.05’ ‘-mind 0.2’ options. Eurasian populations were selected resulting in 938 present-day genome samples for the final visualization. Principal component analysis (PCA) was performed using EIGENSOFT (v6.1.4) (Kang et al. 2010) with default settings. The output was plotted in the R program (Team 2018) (v. 3.5.1) using the ggplot2 (Wickham 2009) (v3.1.0), data.table (v1.11.8) (https://CRAN.R-project.org/package=data.table), grid (v3.5.0) (https://cran.r-project.org/src/contrib/Archive/grid/) and gridExtra (v2.3) (https://cran.r-project.org/web/packages/gridExtra/index.html) packages.

We performed phylogenetic analysis to check the concordance with the aforementioned PCA analysis. We selected 36 Eurasian ethnic groups with available SNP data from HOSP (Lazaridis et al. 2014). For phylogenetic tree construction, we calculated pairwise nucleotide distances (pi) and constructed a neighbor-joining tree using Mega 7 (Kumar et al. 2016). The phylogenetic tree and the map building were conducted using the ggplot2 (v3.1.0) (Wickham 2009), ggtree (v3.8) (Yu et al. 2016), ggpubr (v0.2) (https://rpkgs.datanovia.com/ggpubr/index.html), and data.table (v1.11.8) (https://CRAN.R-project.org/package=data.table) packages of R (v3.5.1) (Team 2018). Longitude and latitude information with custom color and shape settings were used to visualize the physical distances between MJS and other population samples.

### Admixture *f3* statistics based on ancient and present-day genomes

We inferred MJS’s genetic lineage using admixture *f3*-statistics, a method based on measuring the allele frequency correlations between populations (Patterson et al. 2012). To maximize the comprehensiveness of this analysis and evaluate genetic associations, we selected 2670 present-day and 108 ancient genomes (using published ancient genomes (Lazaridis et al. 2014; Lipson et al. 2018; Keller et al. 2012; Jones et al. 2015; Siska et al. 2017; Haak et al. 2015; Allentoft et al. 2015; Skoglund et al. 2014; Fu et al. 2014; Raghavan et al. 2014; Seguin-Orlando et al. 2014; Gamba et al. 2014)). To measure genetic associations, we used a notation *f3*(A,B;Kazakh), where A and B were ancient and present-day populations in various combinations. We employed the qp3PopTest program (v300) from the ADMIXTOOLS (v3) package to calculate the *f3* statistics. We extracted the combinations which had high significance (|Z| > 3) and a sufficient number (> 1000) of examined SNPs (Table S2). The top 30 genome pairs with the most negative *f3* values are plotted (“Results and discussion” section).

### Sequentially Markovian coalescent analyses

Pairwise sequentially Markovian coalescent (PSMC) analysis was conducted to predict and visualize the level of genome diversity (Li and Durbin 2011). To estimate the historical effective population size (*N*_e_) of the Kazakh genome, we applied the PSMC model, using one diploid genome per population and the MSMC2 program (de Manuel et al. 2016). We calculated *N*_e_ for Kazakh (MJS) representing Central Asia and eight additional genomes representing the Northeast and South Asia (Han, Korean, Mongolian, Koryak and Pathan), Africa (Bot San), Europe (French) and the Near East (Turkish), obtained from the PAPGI dataset (http://papgi.org).

Multiple sequentially Markovian coalescent (MSMC) analysis was carried out using the MSMC2 program (de Manuel et al. 2016) to estimate coalescence rates between the haplotypes of the population through time (Schiffels and Durbin 2014). For comparison, we used a dataset from the PSMC analysis containing one diploid genome per population from the PAPGI data. We estimated the depth of coverage of each chromosome (except for the sex chromosomes and mtDNA) from BAM files and, then, we ran SAMtools (Li et al. 2009) to generate mask bed files and VCF files. The script generate_multihetsep.py was used to generate input for the MSMC tool, which merges VCF files. Input files denoting chromosome numbers and positions of segregating sites were generated from all the somatic chromosomes. The results were plotted using R (Team 2018). We adjusted the outcome by setting a mutation rate of 0.5 × 10^−9^ bp^−1^ year^−1^ as suggested by the work on human evolution by Scally (Scally 2016).

## Results and discussion

### Genome sequencing statistics

In total, 82.49 Gbp of nucleotide sequence was generated and mapped to the Human Reference genome (build 37) using BWA (Li and Durbin 2009). We successfully aligned 99.96% of the reads to the reference genome with an average sequencing depth of 29-fold (Table S3). By comparing the MJS genome with the reference, we observed 5,063,461 short variations that consisted of 4,301,702 single nucleotide variants (SNVs) and 761,759 insertions and deletions (indels). MJS mitochondrial genome was mapped by 100% amplicon coverage (16,569 bp), and contained 32 variants including one novel deletion in the 16S ribosomal DNA and variants associated with Leber’s hereditary optic neuropathy (Table S4).

**Fig. 1:**
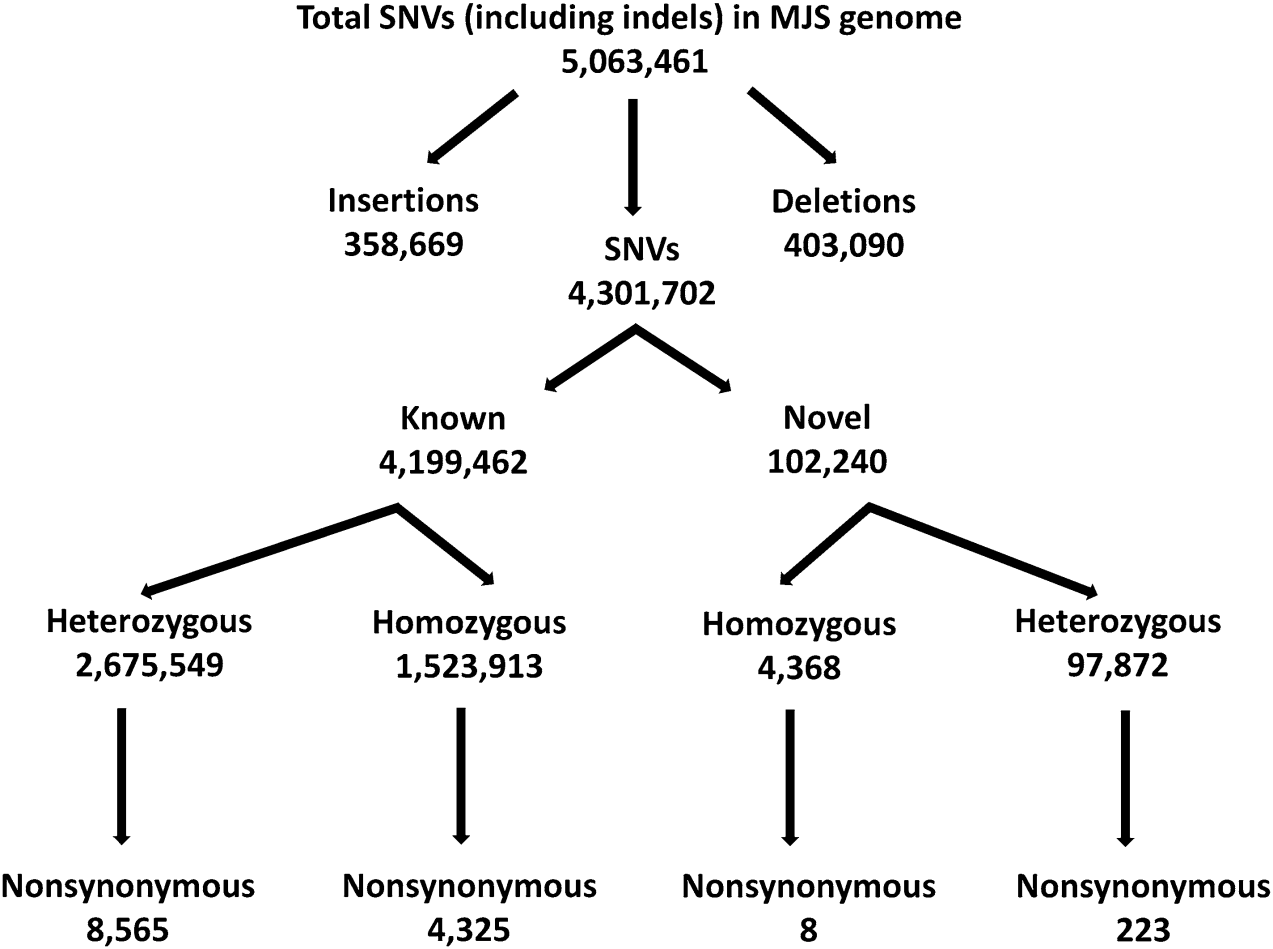
Classification of short variants found in the MJS genome

A total of 4,199,462 SNVs (97.6%, excluding indels) from the whole genome sequence were known variants already deposited in dbSNP (ver. 147), and 102,240 were novel (Fig. 1). In addition, 231 of the novel SNVs (0.2%) were non-synonymous (nsSNVs).

The number of observed heterozygous SNVs in MJS (2,773,421) was compared with that of 212 whole genomes represented in the PAPGI data to visualize levels of heterozygosity per continental group. We observed a significantly higher level of heterozygosity in MJS than in American, Oceanian, East and Southeast Asian genomes, and moderately higher than an average heterozygosity value of the North, South and Central Asian individuals. Still, heterozygosity was lower than the African genomes used in this analysis (Fig. S2).

### Functional classification of the variants

There were 13,121 (0.3%) non-synonymous SNVs (nsSNVs) in MJS genome, of which 5001 (38%) nsSNVs were predicted to be deleterious by SIFT (Ng and Henikoff 2003), Polyphen2 (Jordan et al. 2011), or PROVEAN analysis (Choi and Chan 2015). Out of the 5001 nsSNVs, 654 (12.5%) were identified as deleterious by all of the three prediction methods (Table S5 for known SNVs, Table S6 for novel SNVs). Most of these potentially deleterious nsSNVs (572) were heterozygous, and did not result in any known pathological phenotypes in MJS, suggesting they are functionally benign. The ClinVar (Landrum et al. 2014) (ver. 20170130) analysis of MJS identified 50 SNVs as pathogenic (Table S7). Among them, we found an SNV in the *SCN5A* gene (rs1805124) reported to have a strong link to cardiovascular failure (Mazzaccara et al. 2018). The leading cause of death in Kazakh population is ischaemic heart disease, which caused 32.5% (51.4 thousand) of all deaths in 2012 (WHO 2015) and nearly half of the collected Kazakh genomes (10/22) had at least one (rs1805124) risk variant (Table S1).

Among the drug response variants (Table S8) three important mutant alleles (NAT2*5B, NAT2*6A, NAT2*12A) of slow acetylation activity in the liver were identified in *NAT2* gene (Vatsis et al. 1991) in MJS and one more sample (Table S1). Even though the pathogenicity of these variants isn’t clear (Table S8), these variants were expected to be found in our data set, since they reflect the long history of agriculture in Central Asian populations (Magalon et al. 2008). Also, in 2008, Magalon et al. (Magalon et al. 2008) reported that Kazakh population contains 26–35% of slow acetylators and the haplotype NAT2*5B exhibited the highest allele frequency among Kazakhs, while NAT2*6A was found to be approximately three times more commonly in the neighboring Tajik population.

The MJS metabolism of anticoagulant and anticonvulsant medicines, such as mephenytoin, warfarin, tolbutamide, and phenytoin may be compromised due to the pathogenic mutations in *CYP2C19* (rs4244285) (Arici and Özhan 2017) and antiepileptic carbamazepine metabolism affected by rs1051740 polymorphism (which was predicted to be deleterious by all the three previously described functional impact prediction tools) in the gene *EPHX1* (Zhao et al. 2019). Moreover, substitution of the valine to alanine found in MJS in position 174 of the *SLCO1B1* gene (rs4149056), which was also confirmed by multiple tools to be deleterious, is known to reduce uptake and transport activity of cholesterol-lowering drugs such as simvastatin, pravastatin, pitavastatin, and fexofenadine (Voora et al. 2009) which increases the risk of statin-induced myopathy (Link et al. 2008). This risk variant was found in one more Kazakh sample (2/4, Table S1). Finally, two homozygous variations in MJS in *TAS2R38* (rs10246939 and rs713598), while heterozygous in other Kazakhs, confirmed phenylthiocarbamide taster phenotype (4/4); the ability to taste bitterness in foods, like cabbage, raw broccoli as well as in the drinks like coffee and beer (Perna et al. 2017). Overall, MJS and the additional genomes presented not only well-established variants in the region but also potential for various pharmacogenomic research directions that could be relevant for Kazakh population.

### Hints of Caucasian admixture

MJS has both T and C alleles in the *EDAR* gene (Table S1). In Fujimoto’s research in 2008, out of 360 alleles from Japanese and Chinese samples, 87.6% of them were C, whereas the frequency of the C allele occurrence among European samples was 0% (Fujimoto et al. 2008). The C allele (rs3827760), a hereditary determinant of increased hair thickness, occurred in East Asia, likely in Central China around 30,000 years ago (Kamberov Yana et al. 2013). Just four out of 22 Kazakh samples demonstrated all possible genotypes suggesting that hair thickness in Kazakh population range from the typical (increased) in East Asia to typical in Europeans (Table S1).

MJS also has heterozygous ancient 111T and 374F alleles (rs1426654 and rs16891982) in *SLC45A2* and *SLC24A5* genes that account for the skin tone, as well as eye and hair color, which are nearly fixed in Europeans and, therefore, are ancestry-informative (Soejima and Koda 2007). Moreover, the SNV in the *ABCC11* gene resulted in wet earwax in MJS; the same phenotype can be inferred from all the other Kazakh sequences as well (4/4). Commonly, in East Asian populations the homozygous 180Arg allele is associated with dry earwax, whereas all Europeans have the 180Gly allele that results in wet earwax (Yoshiura et al. 2006). The heterozygosity of the above-mentioned SNVs suggests that the Kazakh MJS genome has genetic overlaps with the European and Asian phenotypes. The assumption of Caucasian admixture was also supported by mtDNA haplogroup J1c2 found in the MJS which is primarily found in Near Eastern and European populations (Hartmann et al. 2009). Even though Kazakh population contains both East Eurasian (55%) and West Eurasian (41%) mtDNA lineages (Berezina et al. 2011), J haplogroup is observed in only 3.6% of the Kazakhs (Berezina et al. 2011). The haplogroup J1c2 is strongly associated with the earliest European farmers and, in fact, has been recently (2017) traced back to the Iron Age Black Sea Scythians (Juras et al. 2017), which points to a European or Near Eastern maternal lineage as a component of the presumably ancient admixture in MJS. However, such genetic affinities may also be indirect, e.g., involving ancestors of ancient nomadic Turkic people whose direct influx into Kazakh lands occurred much later, in the Common Era (West 2009).

### Genetic diversity and structure

**Fig. 2:**
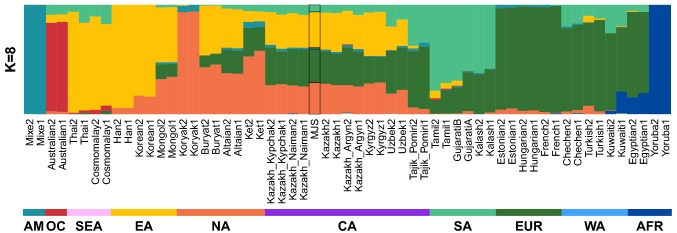
ADMIXTURE plot showing the MJS genome originating from multiple different artificial ancestral populations. Each modern population is represented by genomes which are depicted as colored bars. Each color indicates a different ancestral group and proportion of possibly shared ancestry within larger subgroups.

We employed the ADMIXTURE (Alexander et al. 2009) program to estimate possible ancestries of the MJS genome based on autosomal SNPs (Figs. 2, S3). At *K* = 8, major genetic components were East Asian (yellow) (32.8%), followed by European (dark green) (30.8%) which was also shared by West Asian ancestries as well as Tajik from the Central Asia (Fig. 2, Table S9). The third major MJS component (orange) was attributed mainly to North Asia (28.9%). Around 6% of the MJS’s genome was associated with South Asians (light green portion). The MJS admixture model was tested by qpGraph and it confirms that Europeans and the mixture of North and East Asians were the best fitting admixture sources (Fig. S4). Furthermore, MJS’s ancestral composition was very similar to that of the other Kazakhs used in the dataset, despite of fairly large geographical distances among the sample origins within the country (Materials and Methods 2.4) and different tribal affiliations. Comparing to other Kazakh samples (Kypchak, Naiman, Argyn and mixed Kazakh), MJS genome showed the highest proportion of the North Asian component, which surprisingly varied little among all Kazakh (by 5.6%). However, the European and East Asian portions varied among the Kazakh samples the most—by approximately 8%, followed by the West Asian component (that varied by 4.5%), where in all cases MJS showed quite average proportions (Table S9). We speculate that a high level of heterozygosity (Fig. S2) with very similar ancestral component composition is a common trait of the Kazakhs. It points to a scenario, where Kazakhs experienced admixtures from various different ethnic groups, since ancient times but in recent times kept admixing mainly between the local (tribes) subpopulations leading to a modern Kazakh genetic identity. However, a large-scale study covering statistically significant number of individuals from different regions and covering all tribal lineages is needed to confirm this hypothesis.

### Principal component analysis (PCA)

**Fig. 3:**
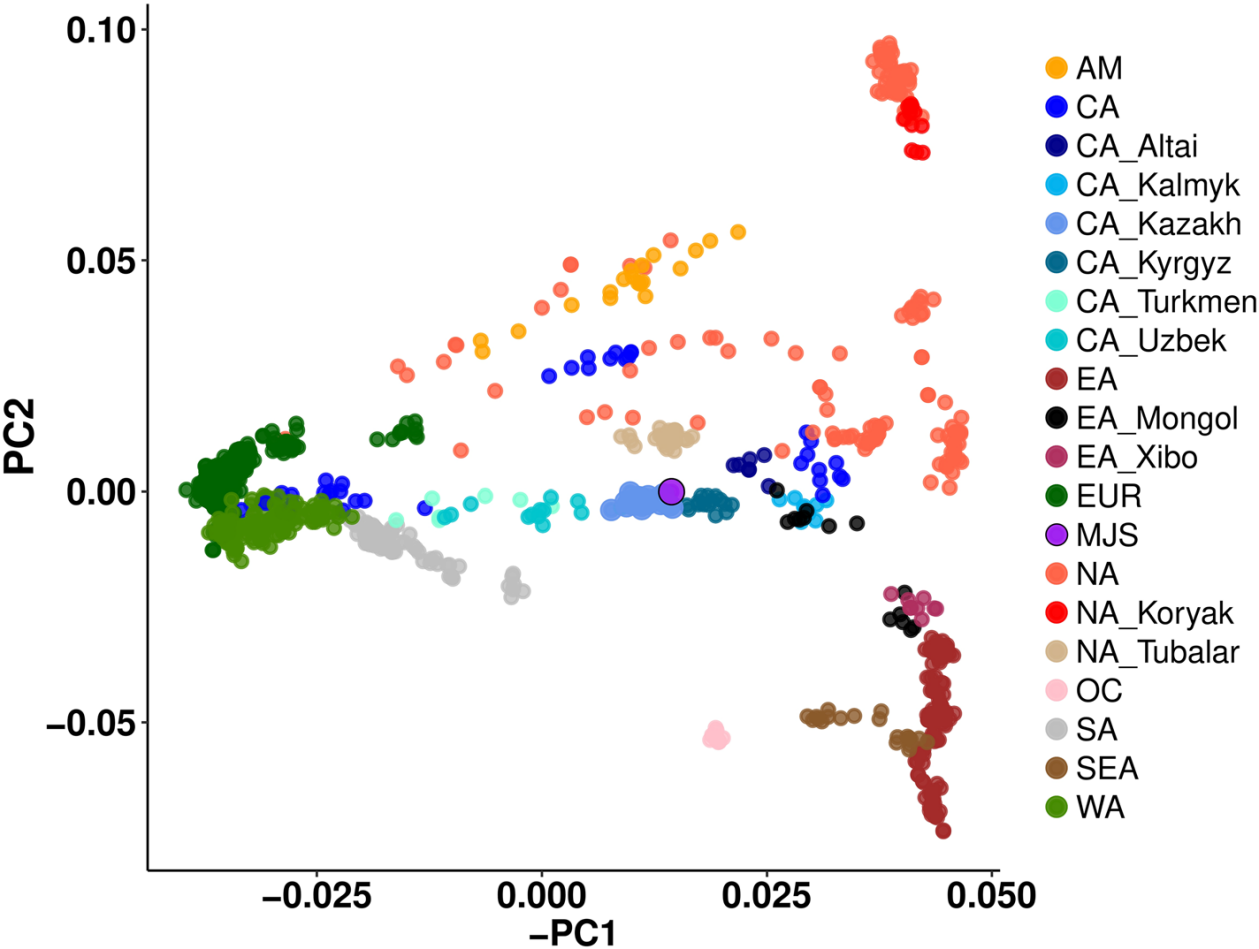
Plot of the first two principal components with the modern Eurasian populations. Dots represent genomes color-coded by the continental groups: Europe (EUR)—dark green, West Asia (WA)—light green, Central Asia (CA)—blue, North Asia (NA)—peach, South Asia (SA)—grey, Southeast Asia (SEA) and East Asia (EA)—brown, and America (AM)—yellow, Oceania (OC)—coral, serving as outgroups. Separate populations are visualized as different shades and the MJS sample is visualized as a purple dot

All Kazakh samples, including MJS, cluster together in a PCA plot and the similarities of samples reflect geographic proximities (Fig. 3). The closest similarity to the Kazakh MJS sample is exhibited by Central Asians (Kyrgyz, Uzbek, Kalmyk) and East Asians (Mongolians), which was confirmed by the phylogenetic tree based on the pairwise nucleotide distances (Fig. S5, Table S10). Moreover, MSMC analysis suggested Kazakh and Mongolian population divergence at around 7000 years ago (Kya) which means relatively recent common ancestry compared to divergence of the Kazakh and Koryak or Kazakh and Han Chinese (around 10 Kya) (Fig. S6). The Near Easterner (Turkish) population separation was estimated to have occurred prior to 13,000 years ago (Kya) (Fig. S6). Although MJS genome has high heterozygosity, all of the methods employed hint higher Kazakh (including MJS) genetic affinity to East Asians (Mongolian) than Caucasians as previously reported (Tarlykov et al. 2013).

The Kyrgyz appeared to be the closest population to the Kazakhs. It is widely speculated that the Naiman tribe, the paternal line of MJS, has close ethnogenetic ties with the Yenisei Kyrgyz and once coexisted together under the Kyrgyz Khaganate (Akerov 2016). The split between the two might have occurred around the fifteenth–sixteenth century CE, along with Kyrgyz migration (Heyer et al. 2009). The Kalmyk similarity to MJS is not surprising as in the seventeenth century CE Kalmyks conquered Western Mongolia as well as Eastern and South Eastern Kazakhstan. Their territories continued to expand at the cost of Kazakh lands until the eighteenth century CE (Olcott 1995) which might have resulted in the direct admixture of the Kazakh and the Kalmyk despite already shared genetic influences from the Mongolian empire (Nasidze et al. 2005). As for Uzbek, their genomic similarity can historically be traced back to the Uzbek Khanate in the fifteenth century CE as they have coexisted with many other tribes including the Dulat (ancestors of MJS’s maternal lineage) in various tribal groupings until the two populations split (Paksoy 1992). Altaians (Tubalars) appeared to be genetically closest ethnic group from the North Asia (Table S10). Besides their close geographic proximity and shared roots (Dulik Matthew et al. 2012), previous research suggests a significant demographic expansion of Altaian people from the Mongolian Altai territories towards Western steppes after the seventh century CE, based on the Iron Age Western Altaian (Russian and Kazakh territory) and Mongolian Altaian mtDNA similarities (Dulik et al. 2011). On the other hand, a wave of Kazakh migration around the Altai mountains occurred around the nineteenth–twentieth century CE (Krader 1966), likely from the Middle Zhuz (Oktyabrskaya 2006), from which MJS’s paternal line originates. Some of the destinations of this fairly recent migration were as far as Xinjiang in China, and Western Mongolia (Krader 1966). This migration seems to be surprisingly accurately depicted in the MJS genome as both Mongolian and Xibo tribe (residing in Xinjiang) are two of the most closely related East Asian populations to MJS (Table S10, Fig. S5). A relatively recent Y-chromosome variation analysis proposed shared Kazakh paternal lineages with the Mongolian and attributed their findings to the Mongolian Empire expansion in the thirteenth century CE (Dulik et al. 2011). The Mongolian genetic influence in the MJS genome also can be traced to her paternal ancestors, members of the Naiman tribe.

### Admixture *f3* statistics based on ancient and present-day genomes

**Fig. 4:**
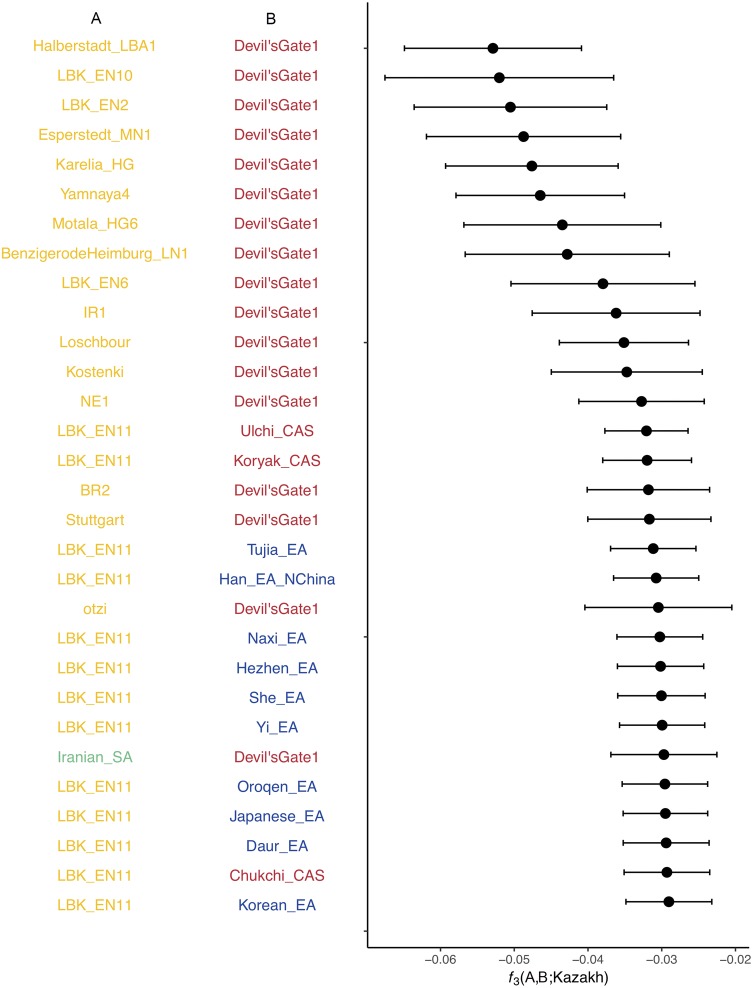
The Admixture *f3* analysis representing MJS genome as a mixture of genomes A and B. The genomes are color-coded by regions; yellow representing ancient genomes found in Europe, red—ancient genomes from Northeast Asia and indigenous (present-day) genomes from Russian Far East, blue—present-day East Asian populations, and green—present-day West Asian populations. Thirty pairs with the lowest *f3* score for the Kazakh (MJS) are presented

Knowing the complex Kazakh history which is rich of different admixture sources, we used admixture *f3* statistics to test if it is possible to define a present-day Kazakh individual, MJS, as an admixture product of only two distinct populations (Sudmant et al. 2015) and what pair of populations would have the highest similarity. Even though the pairwise allele sharing was measured with both present-day and ancient genomes, the highest genetic affinity in nearly all 30 cases was shown by a pair of ancient genomes, where one ancient genome comes from Europe and one from Northeast Asia (Fig. 4). From the Asian genomes the highest similarity was shown by the Devil’s gate sample Devil'sGate1, an Early Neolithic hunter-gatherer (Siska et al. 2017); it appeared in more than 10 best representing pairings. So far, the Devil’s gate genomes (1 and 2) are the closest to East Asia genomes available today (Siska et al. 2017). Moreover, the present-day populations from China (Tujia, Han, Naxi, She, Hezhen, Oroqen, Daur and Yi) and other East Asian territories (Japanese and Korean) consistently showed some of the highest genetic affinities with Kazakh (MJS) supporting the theory of the genetic continuity within the East Asian region (Siska et al. 2017) and strongly reflecting the East Asian component in MJS genome. The ancient European genomes with the highest proportion of allele sharing were excavated from the Central Europe; the Halberstadt_LBA1 genome dated Late Bronze Age, LBK_EN samples attributed to Early Neolithic and Esperstedt to Middle Neolithic periods (Haak et al. 2015). The overlap in time frame (Early Neolithic) of the Devil’s gate and Central European genomes suggests the presence of two possible ancient genomic components of different origins present in the MJS. Interestingly, Kazakh effective population size *N*_e_ prior to the Neolithic era has undergone a radical decrease; around 60,000 years ago (Kya), which would correspond to Middle Paleolithic (Bicho 2013), Kazakh *N*_e_ reached its lowest—less than 4000 individuals and around the Upper Paleolithic (Klein 1999) (40 Kya) recovered to approximately 6000 (Fig. S7). The end of the Last Glacial Maximum and subsequent population size increase suggest it became possible for ancestral MJS’s populations of different origins to migrate and admix at around that time.

Even though other ancient European genomes in this analysis were attributed to different locations and times; ranging from the Holocene (Lazaridis et al. 2014; Haak et al. 2015) to Iron Age (Gamba et al. 2014), the strong MJS’s allele frequency association with ancient Europeans prevails. Moreover, the only present-day population paired with the East Asian genome (Devil’s gate) is Iranian, which strengthened the evidence of a European/Near Eastern component as inferred by the MJS mtDNA haplogroup. These two components, however, may not be the direct or the only sources of admixture in MJS and other Kazakh as such model does not estimate demographic shifts and complex admixtures from multiple sources.

## Conclusions

We present the whole genome sequence and thorough genetic variant and admixture analysis of a Central Asian, Kazakh MJS. We found several SNVs associated with drug toxicity, metabolism, diseases, phenotypic features and identified recent and ancient admixtures. Both PCA and phylogenetic analyses confirm closer MJS and other Kazakh similarity to modern East Asians than Europeans and showed the overall closest genetic affinities are with other Central Asian populations, namely, Kalmyk, Uzbek and Kyrgyz. All populations with significant similarity to MJS genome could be backed up by historic migration events involving the Kazakh population and the major fraction of genomic variation could be attributed to fairly recent admixture with geographically close populations. However, MJS’s mitochondrial DNA haplogroup is of European or Near Eastern (West Asian) ancestry. It corresponds to the heterozygous SNPs associated with European phenotypic features and confirmed by admixture *f3* statistics and all other Kazakh autosomal data showed very similar ancestral compositions to MJS’s. This highly heterozygous and admixed Kazakh genome provides insights into complex admixtures and can serve as a reference for mapping complex heterogeneity in Central Asian populations.

## Electronic supplementary material

Below is the link to the electronic supplementary material.

**Fig. S1.** The cross validation error values in ADMIXTURE in relation to *K.* (TIFF 43 kb)

**Fig. S2.** Heterozygous to homozygous SNP ratios of genomes from the PAPGI dataset. Genomes were grouped into boxplots by their continents. Red line indicates the ratio of MJS. (TIFF 389 kb)

**Fig. S3.** An ADMIXTURE plot showing the increasing complexity of MJS genome as the number of artificial ancestral groups increases from *K* = 2 to K = 8. Each sample is represented by a colored bar. The colors within the bars indicate possible ancestral groups. Shared colored fractions among the samples indicate shared artificial ancestry. (PDF 15 kb)

**Fig. S4.** The qpGraph results of four Kazakh admixture models. The dashed lines represent admixture events tested and the percentages denote the proportions of admixture relative to the two admixture sources. Units along the solid lines represent the measure of drift. The Z-scores are − 2.918, − 2.794 for panels A, B, respectively, and − 2.617 for panels C and D. (JPEG 3610 kb)

**Fig. S5.** The phylogenetic relationship of MJS and various populations. A) Phylogenetic tree based on pairwise nucleotide distances between the MJS and other population samples. B) Geographical location of samples used in the phylogenetic tree construction (TIFF 2040 kb)

**Fig. S6.** Relative cross-coalescence rate over time showing the genomic diversification history of the Kazakh individual (MJS) (TIFF 1089 kb)

**Fig. S7.** The PSMC analysis showing retrospective changes in effective population size *N*_*e*_ of the Kazakh (MJS). The effective population size *N*_*e*_ of the Kazakh is based on MJS genome, plotted together with *N*_*e*_ of African (Bot San), Northeast and South Asian (Han, Korean, Mongolian, Koryak, and Pathan), European (French), and Middle Easterner (Turkish) genomes. (TIFF 1146 kb)

**Table S1:** Pathogenic and phenotypic variant analysis in the Kazakh based on the findings in MJS genome. **Table S2:** Z and *f3* values of MJS in an admixture *f3* test. **Table S3:** Mapping statistics of MJS genome. **Table S4:** A list of the variants in MJS's mtDNA. **Table S5:** A list of non-synonymous known deleterious SNVs confirmed by SIFT, Polyphen2, and PROVEAN prediction methods. **Table S6:** A list of non-synonymous novel deleterious SNVs confirmed by SIFT, Polyphen2, and PROVEAN prediction methods. **Table S7:** SNVs predicted to be pathogenic in ClinVar database (v20170130). **Table S8:** SNVs related to drug response in ClinVar (v20170130). **Table S9:** Kazakh genomes ADMIXTURE components. **Table S10:** Phylogenetic distance score matrix of genomes (XLSX 1104 kb)

## Data Availability

The MJS’s whole genome sequence analyzed in this study has been deposited in the NCBI SRA database under accession No. SRS2904218 and NCBI BioSample database under accession No. SAMN08442411. Other datasets are currently available from the corresponding author on reasonable request. Datasets in this study were made using publicly available resources such as PAPGI, EGDP, HOSP and previous studies described in detail in the methods section for each analysis.
