# Supplementary figures and images for: Decoding a highly mixed Kazakh genome
Source: Hum Genet. 2020 Feb 19;139(5):557–68. doi: 10.1007/s00439-020-02132-8 (PMC7170836; doi:10.1007/s00439-020-02132-8)

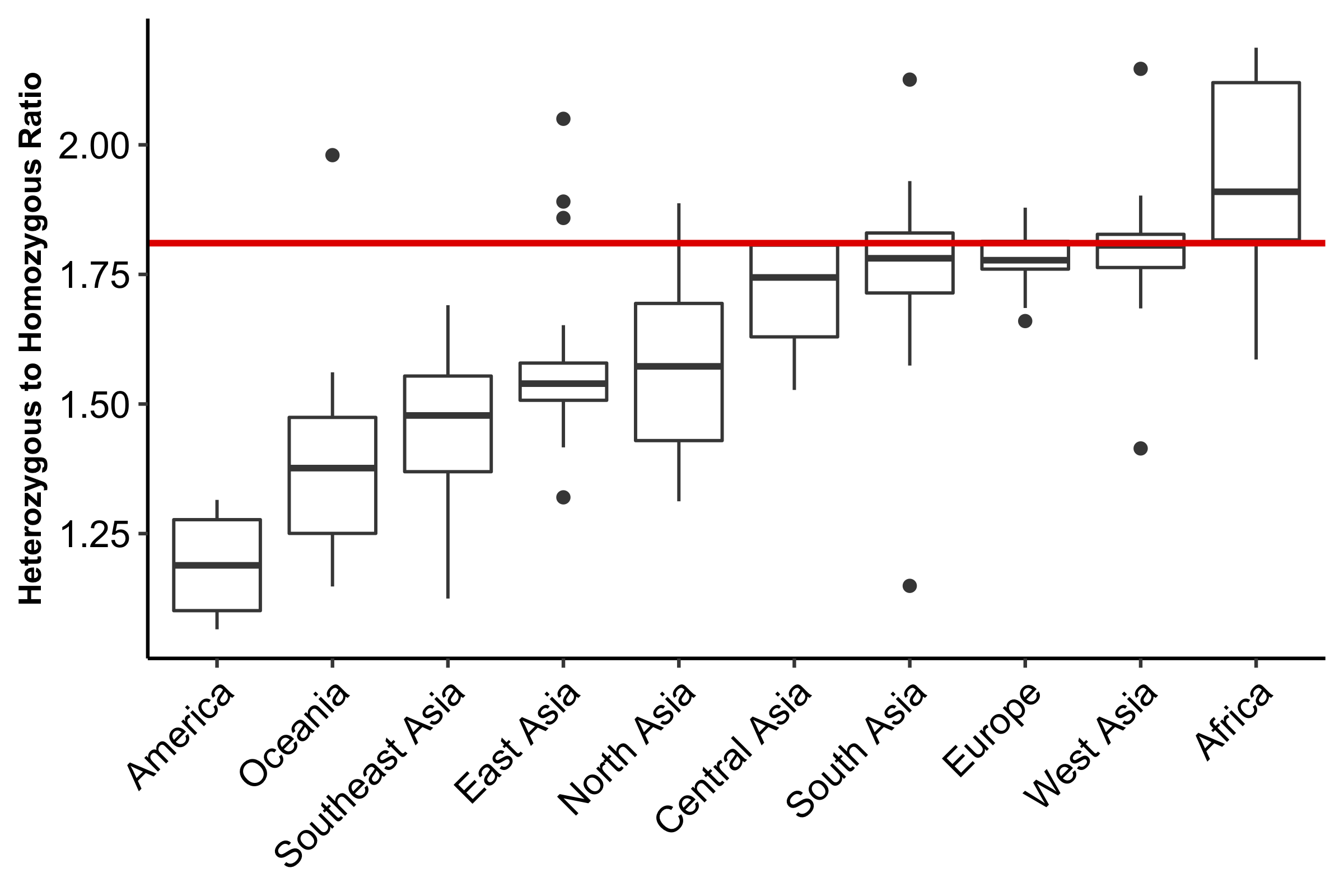

Supplement: Supplementary file 2 — Fig. S2. Heterozygous to homozygous SNP ratios of genomes from the PAPGI dataset. Genomes were grouped into boxplots by their continents. Red line indicates the ratio of MJS. (TIFF 389 kb) [file 439_2020_2132_MOESM2_ESM.tif]

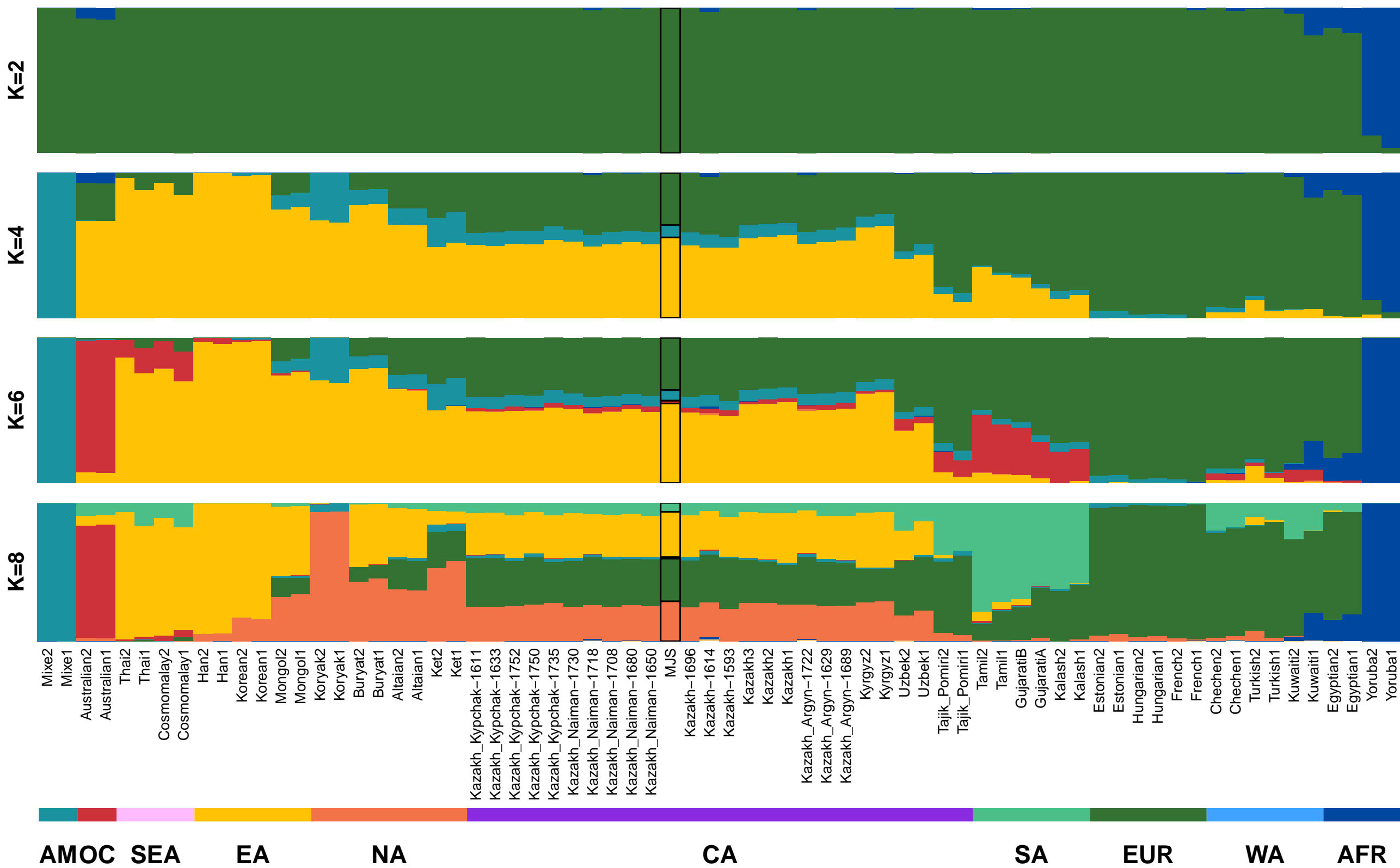

Supplement: Supplementary file 3 — Fig. S3. An ADMIXTURE plot showing the increasing complexity of MJS genome as the number of artificial ancestral groups increases from K = 2 to K = 8. Each sample is represented by a colored bar. The colors within the bars indicate possible ancestral groups. Shared colored fractions among the samples indicate shared artificial ancestry. (PDF 15 kb) [file 439_2020_2132_MOESM3_ESM.pdf]

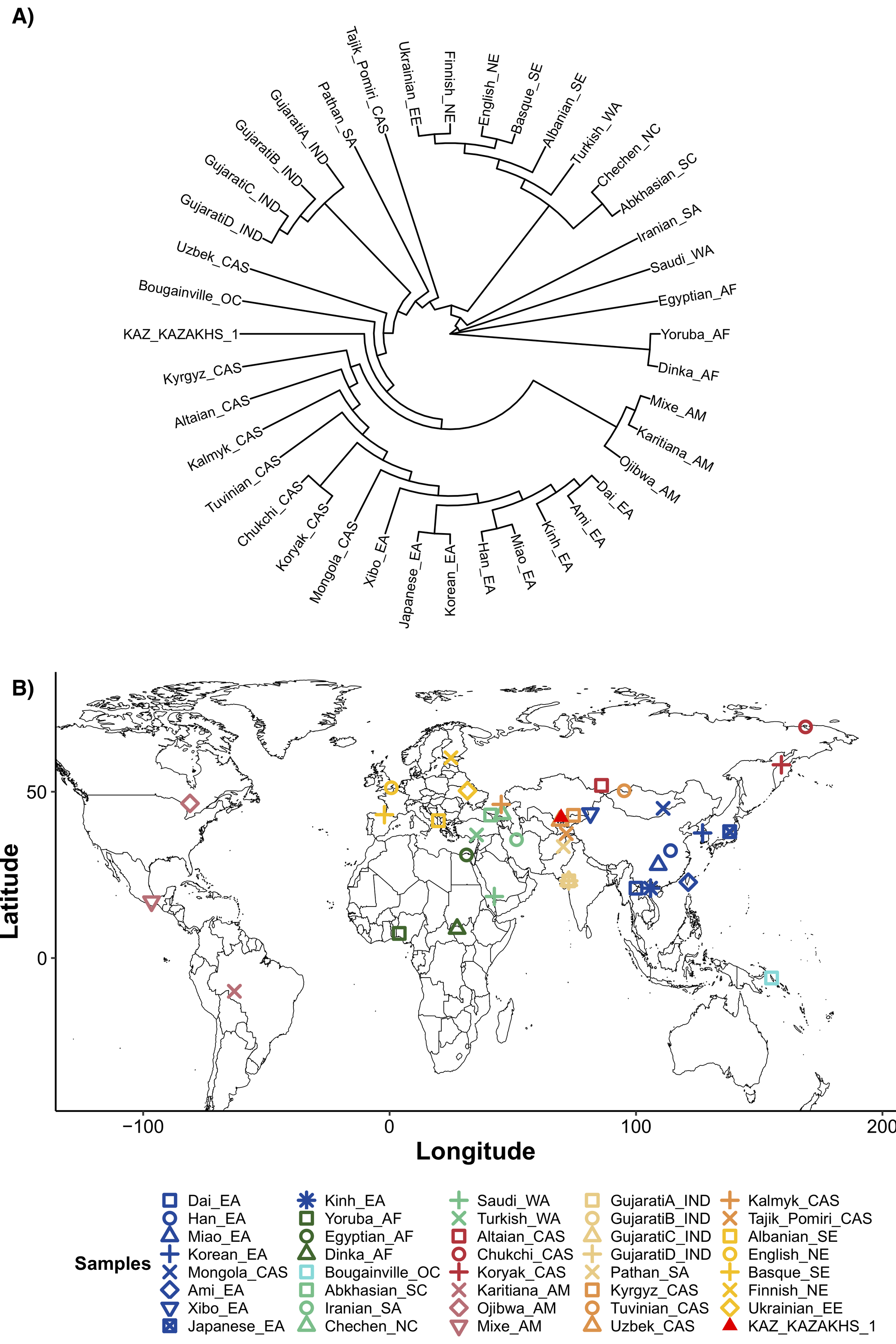

Supplement: Supplementary file 5 — Fig. S5. The phylogenetic relationship of MJS and various populations. A) Phylogenetic tree based on pairwise nucleotide distances between the MJS and other population samples. B) Geographical location of samples used in the phylogenetic tree construction (TIFF 2040 kb) [file 439_2020_2132_MOESM5_ESM.tif]

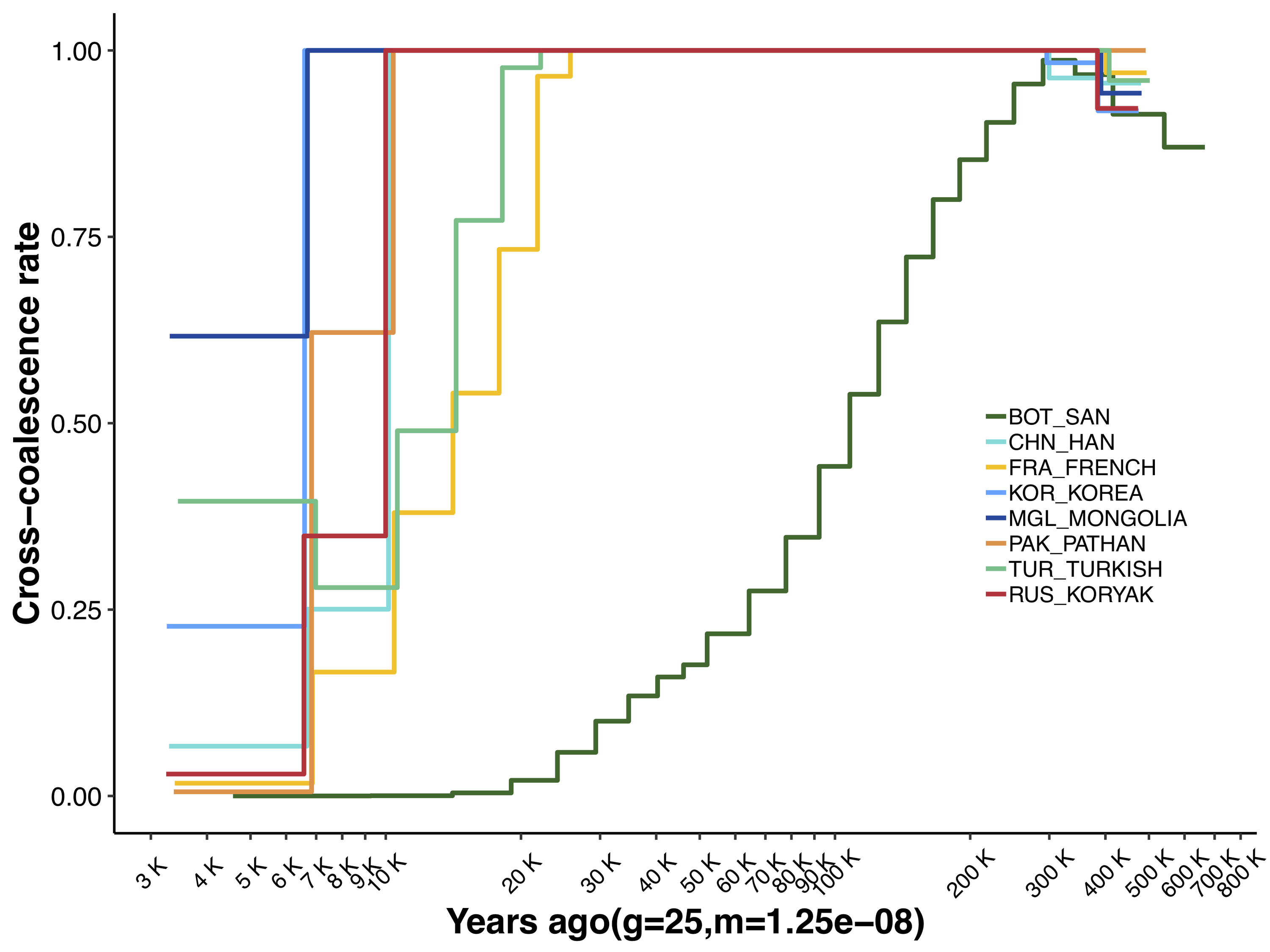

Supplement: Supplementary file 6 — Fig. S6. Relative cross-coalescence rate over time showing the genomic diversification history of the Kazakh individual (MJS) (TIFF 1089 kb) [file 439_2020_2132_MOESM6_ESM.tif]

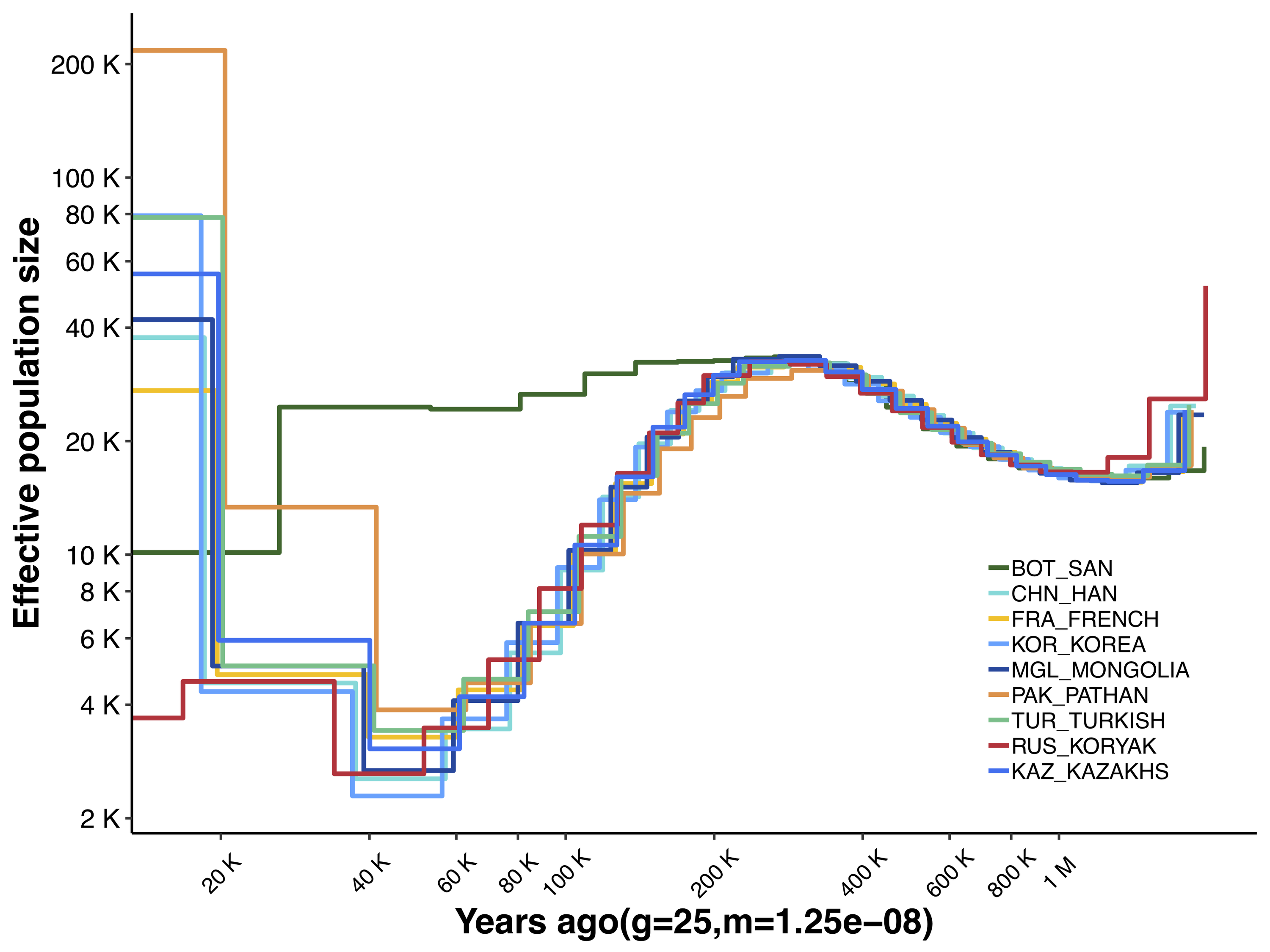

Supplement: Supplementary file 7 — Fig. S7. The PSMC analysis showing retrospective changes in effective population size Ne of the Kazakh (MJS). The effective population size Ne of the Kazakh is based on MJS genome, plotted together with Ne of African (Bot San), Northeast and South Asian (Han, Korean, Mongolian, Koryak, and Pathan), European (French), and Middle Easterner (Turkish) genomes. (TIFF 1146 kb) [file 439_2020_2132_MOESM7_ESM.tif]
